# Substance P and Calcitonin Gene-Related Peptide: Key Regulators of Cutaneous Microbiota Homeostasis

**DOI:** 10.3389/fendo.2017.00015

**Published:** 2017-01-30

**Authors:** Awa N’Diaye, Andrei Gannesen, Valérie Borrel, Olivier Maillot, Jeremy Enault, Pierre-Jean Racine, Vladimir Plakunov, Sylvie Chevalier, Olivier Lesouhaitier, Marc G. J. Feuilloley

**Affiliations:** ^1^Laboratory of Microbiology Signals and Microenvironment (LMSM), Normandie Université Rouen, Evreux, France; ^2^Winogradsky Institute of Microbiology, Research Center of Biotechnology of Russian Academy of Science, Moscow, Russia

**Keywords:** skin bacterial communication, substance P, calcitonin gene-related peptide, DnaK chaperone protein, EfTu thermo unstable ribosomal elongation factor, MscL mechanosensitive channel, moonlighting proteins, microbial endocrinology

## Abstract

Neurohormones diffuse in sweat and epidermis leading skin bacterial microflora to be largely exposed to these host factors. Bacteria can sense a multitude of neurohormones, but their role in skin homeostasis was only investigated recently. The first study focused on substance P (SP), a neuropeptide produced in abundance by skin nerve terminals. SP is without effect on the growth of Gram-positive (*Bacillus cereus, Staphylococcus aureus*, and *Staphylococcus epidermidis*) and Gram-negative (*Pseudomonas fluorescens*) bacteria. However, SP is stimulating the virulence of *Bacillus* and *Staphylococci*. The action of SP is highly specific with a threshold below the nanomolar level. Mechanisms involved in the response to SP are different between bacteria although they are all leading to increased adhesion and/or virulence. The moonlighting protein EfTu was identified as the SP-binding site in *B. cereus* and *Staphylococci*. In skin nerve terminals, SP is co-secreted with the calcitonin gene-related peptide (CGRP), which was shown to modulate the virulence of *S. epidermidis*. This effect is antagonized by SP. Identification of the CGRP sensor, DnaK, allowed understanding this phenomenon as EfTu and DnaK are apparently exported from the bacterium through a common system before acting as SP and CGRP sensors. Many other neuropeptides are expressed in skin, and their potential effects on skin bacteria remain to be investigated. Integration of these host signals by the cutaneous microbiota now appears as a key parameter in skin homeostasis.

## Introduction

Skin is a complex ecosystem hosting the second most numerous microbial population of the human body (1). Skin-associated bacteria, which represent the essential of this population, are classically divided into two categories, i.e., commensal and transient germs. While commensal bacteria are considered having protective functions, transient ones include opportunistic pathogens. However, this reductive vision is far from reality and germs such as *Staphylococcus aureus*, originally considered as pathogens, are chronically carried by 35–60% of the human population (2). Other bacterial pathogens, such as *Bacillus cereus, Pseudomonas aeruginosa* or even *Neisseria meningitidis*, can also be encountered on normal skin in a total absence of clinical signs (3). In fact, virulence is not a constant trait for the large majority of the bacterial species, and expression of virulence is closely controlled by bacterial communication designated under the general term of quorum sensing (QS) (4). The QS is triggered in Gram-negative bacteria by *N*-acyl homoserine lactones and in Gram-positive bacteria by cyclic or linear peptides. Sensing these molecules can lead pathogenic, but also non-pathogenic, bacteria to switch from harmless to aggressive by regulating the production of most of their virulence factors. However, communication is not solely based on intra- or interspecies bacterial QS, since bacteria also need adapting to their host, thereby sensing numerous eukaryotic signals, including skin neuropeptides. These mechanisms are acknowledged to be part of interkingdom communication.

Skin is the principal neuroendocrine organ of the human body (5), and since 1930 it is known that neurotransmitters such as catecholamines can modulate bacterial infection (6). However, at this time, bacteria were considered inert, and this observation was attributed to an unknown effect of adrenalin on the human physiology. It was necessary to wait for the work of Lyte and Ernst in 1992 to show that catecholamines can promote the growth of *Escherichia coli* (7) and also bacterial adhesion, biofilm formation, and expression of virulence factors (8, 9). Membrane proteins acting as specific bacterial sensors for catecholamines were identified, and it was then recognized that bacteria can sense human neurotransmitters (10). This concept was extended to neuropeptides and neurohormones, giving birth to microbial endocrinology, which is presently one of the more active fields in microbiology (11). Neuroendocrine factors have multiple effects on bacteria and can regulate their growth, adhesion, invasion, virulence, and/or biofilm formation activity (12). As almost 25% of the microbial population is located deeply into the skin, particularly in hair follicles and sweat or sebaceous glands (13), bacteria are in close contact with eukaryotic cells communication factors. Moreover, it has been shown that neuropeptides diffuse in significant amount in upper epidermal layers and sweat (14, 15). Cutaneous bacteria are then exposed largely to these host factors.

## Effect of Substance P (SP) on Cutaneous Bacteria

In skin, SP is essentially released by sensory primary afferent C-fibers (16). This undecapeptide of the tachykinin family has multiple bioactivities other than neurotransmission, such as fibroblast and keratinocyte proliferation, mast cell degranulation, and capillary vasodilatation (5, 17). It is considered as a major mediator of neurogenic inflammation and has central functions in itch (5). Cutaneous neuropeptides, and particularly SP, are contributing to the pathogenesis of different skin diseases, including psoriasis (18), atopic dermatitis (19), immediate and delayed hypersensitivity (20), acne (21), or rosacea (22). These diseases are of multifactorial origin, and different studies suggest a contribution of the skin microflora but also an involvement of local host factors (23).

### SP Sensing by *B. cereus*

**Figure 1:**
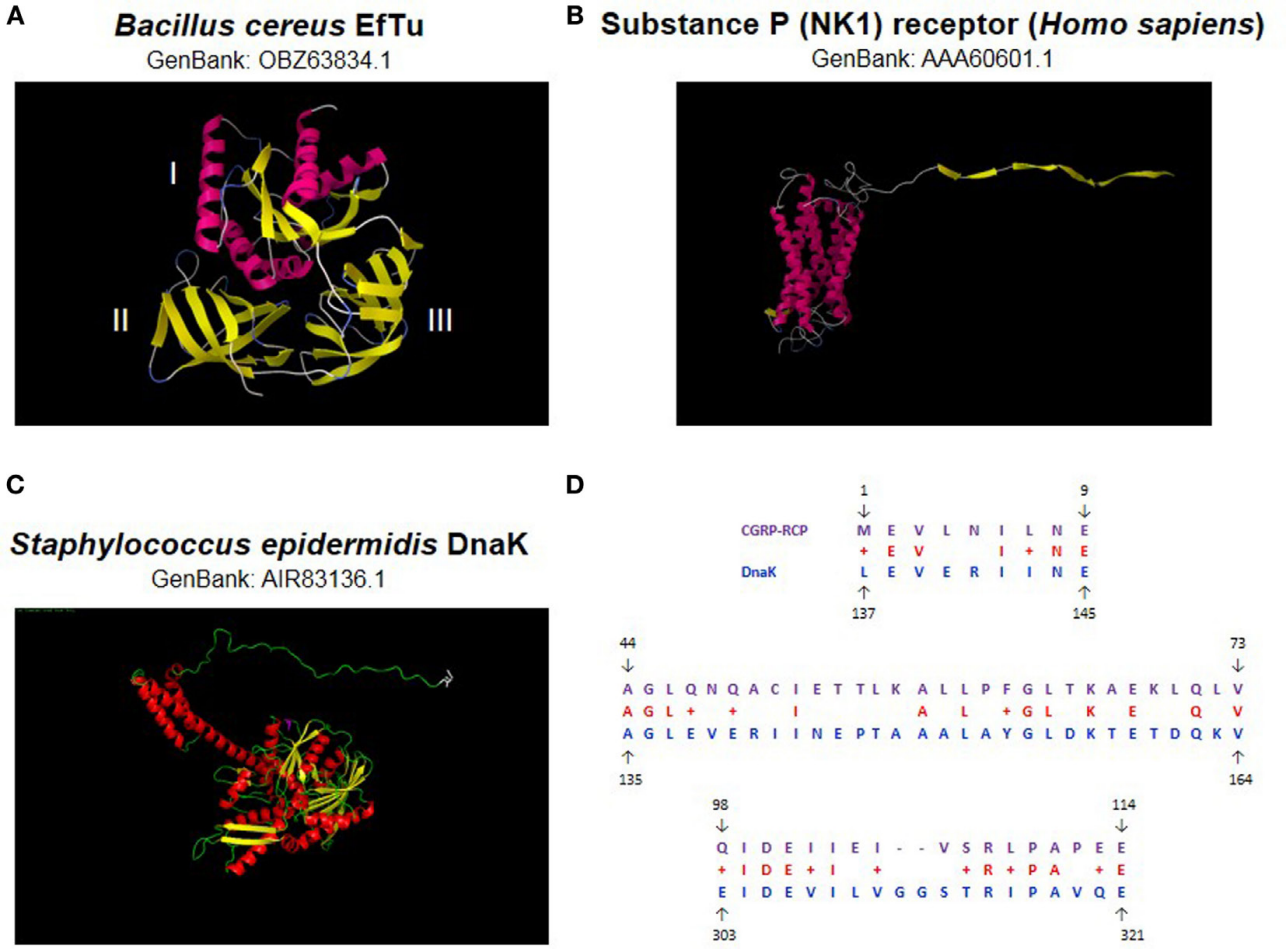
**Bacterial substance P (SP) and calcitonin gene-related peptide (CGRP) sensors**. The thermo unstable ribosomal elongation factor EfTu identified as SP sensor is a 395 amino acids protein (43 kDa) characterized by three principal domains **(A)**. Domain I is essentially organized in alpha helix whereas II and III form beta strands. No signal sequence or transmembrane domain signature is present. EfTu is not showing significant sequence or structural similarities with the NK1 SP human receptor **(B)**. The *Staphylococcus epidermidis* chaperone DnaK identified as CGRP sensor is a 609 amino acids protein (70 kDa) **(C)**. This protein is showing limited similarity with the human CGRP receptor component protein (CGRP-RCP) but higher similarity with an invertebrate (*Ciona intestinalis*) CGRP-RCP (124 amino acids) where DnaK was found covering 45% of its sequence with 40% identity **(D)**. Sequence similarities were investigated by BlastP (https://blast.ncbi.nlm). 3D structures were calculated by RaptorX (31) and visualized using Python Molecular Viewer V1.5.6.

In a first step, the effect of SP was studied on a strain of *B. cereus* (MFP01) isolated from normal human skin (23). Even at a micromolar concentration, SP was without effect on the growth kinetic of *B. cereus*. SP was also without effect of its swimming and swarming modes of motility. However, when *B. cereus* was grown in the presence of SP, the bacterium showed a strong increase of cytotoxicity on HaCaT keratinocytes (24). The threshold of SP activity was nanomolar and therefore in the range of the local concentration of the neuropeptide in skin during inflammation or stress. Bacteria were rinsed to remove any trace of free peptide prior to cell infection, and all the controls realized showed that this effect can only be explained by a direct effect of SP on the bacterium. Moreover, the SP reversed sequence peptide and neurokinin A, which in eukaryotes binds on the same receptor as SP, displayed no activity on cutaneous bacteria (24, 25). *B. cereus* is responding to SP by an over production of collagenase that can explain its effect on HaCaT cells (24). However, a massive cell death, as observed after exposure of keratinocytes to SP-treated bacteria, is associated with the release of high amounts of toxic-oxidizing compounds, and *B. cereus* also reacts to SP by an overproduction of superoxide dismutase (24). This can be considered as a defense reaction of the bacterium. When *B. cereus* was exposed to SP over a long period (13 h), an increase in the release of S-layer proteins was also noted (24). This result was correlated to a peel-off of the S-layer (the outer wall of the bacterium) and to a drop in bacterial surface polarity (24). These events can also be interpreted as a general defense reaction of the bacterium against SP as this peptide has structural homologies with cationic antimicrobial peptides, and S-layer monomers are known to complex and neutralize antimicrobial peptides (26). The response of *B. cereus* to SP being rapid (90.5% of the maximal response in less than 5 min) and the S-layer showing multiple pores allowing the passage of peptides (27), an SP surface binding site was postulated. By using an original immunoprecipitation technique, a 43 kDa protein, the thermo unstable ribosomal elongation factor EfTu, was identified as the SP sensor in the membrane of *B. cereus* (24) (Figure 1A). EfTu is not presenting sequence or structural similarities with the SP (NK1) receptor (Figure 1B). However, it was not surprising that EfTu was acting as a surface receptor on the bacterium because this molecule is present in large excess in bacteria (28), and it is now recognized as a “moonlighting protein” (29). These proteins are characterized by multiple functions in different locations between the bacterial stroma and the membrane. Because of their long evolution and history, bacteria have reached a high level of protein fitting (30); expression of moonlighting proteins should be an alternative strategy to counterbalance the absence of alternative splicing in these organisms.

### SP Sensing by Staphylococci

Further studies revealed that the ability to detect SP is shared by other Gram-positive bacteria such as *S. aureus* and *Staphylococcus epidermidis*. These bacteria react to SP by a marked increase in cytotoxicity and virulence on HaCaT keratinocytes and reconstructed human epithelium (RHE) (25). qRT-PCR arrays revealed that the response of RHE to *S. aureus* and *S. epidermidis* previously exposed to SP is notably different (25). SP-treated *S. aureus* induces an upregulation of CCL5 and CXCL1 chemokines and interleukin 8 expression by RHE suggesting the induction of an inflammatory response. Conversely, SP-treated *S. epidermidis* provoke overexpression of integrin α5 and chemokine ligand 10 CXCL10 (25), two markers of psoriasis (32). Postulating a link between the action of SP on *S. epidermidis* and psoriasis should be a premature conclusion, but this hypothesis is worthy consideration. To get further insights into the role of SP in promoting *S. aureus* and *S. epidermidis* virulence, the effects of the peptide on the adhesion and invasion potential of these bacteria were studied. Exposure of both *Staphylococci* to SP resulted in a marked increase in their adhesion potential on keratinocytes whereas their invasive activities were unchanged (25). Further studies, based on secretome analysis and biofilm formation activities revealed that *S. aureus* and *S. epidermidis* have very different responses to SP. Indeed, although SP has no effect on the release of exoproteins by *S. epidermidis*, this neuropeptide is stimulating the production of staphylococcal enterotoxin C2 by *S. aureus* (25). In addition, in *S. aureus*, SP induces a decrease in the production of lipase, apparently by inhibiting the processing of its precursor (25). Such a metabolic change should favor lipids’ accumulation, and it is well known that a hydrophobic environment is particularly important for the growth of *S. aureus* (33). *Staphylococci* also express the moonlighting protein EfTu and, as in *B. cereus* (24), EfTu was identified as the SP-binding site in *S. aureus* and *S. epidermidis* (25). These results suggest that EfTu should act as an SP sensor in a great variety of Gram-positive bacteria, and potentially also in other eubacteria.

### SP Sensing by Gram-negative Bacteria

In agreement with this hypothesis, it was observed that *Pseudomonas fluorescens*, a typical Gram-negative bacterium and member of the commensal skin microflora (1), is also sensitive to SP (34). In this species, the effect of SP is more limited, as no variation of virulence was detected, but SP was found to increase the adhesion and invasive potentials of the bacterium on HaCaT cells (34). In addition, exposure of *P. fluorescens* to SP is associated with structural rearrangements of the biofilm. Although the SP-binding site was not identified until now in this species, it is interesting to note that, in *Pseudomonas*, EfTu is also known as an environmental sensor (35).

## Effect of Calcitonin Gene-Related Peptide (CGRP) on Staphylococci

Calcitonin gene-related peptide is a neuropeptide abundantly expressed in the skin (9). CGRP is generally co-localized and co-released with SP (36). Two isoforms of this peptide, α-CGRP (or CGRP I) and β-CGRP (or CGRP II), are produced from the same gene by alternative splicing, but α-CGRP is the major form expressed in sensory skin fibers (5). CGRP is known to potentiate the effects of SP on vascular permeability and edema formation. In addition, CGRP exerts trophic effects on endothelial cells and melanocytes. A direct antimicrobial activity of CGRP has been described in *E. coli* and *P. aeruginosa* (37). This effect was explained by structural similarities between CGRP and antimicrobial peptides. However, the antimicrobial spectrum of CGRP is narrow, and this peptide was shown without effect on the viability of *Staphylococci* (37, 38).

### Effect of CGRP on *S. epidermidis* Virulence

When *S. epidermidis* was exposed to CGRP, an important increase of cytotoxicity and virulence was observed on HaCaT keratinocytes and RHE (38). The threshold of CGRP activity on *S. epidermidis* is remarkably low (<10^−12^ M) suggesting a very specific action. Conversely, no effect of CGRP was found in *S. aureus* (38). As in nerve terminals CGRP is co-secreted with SP, *S. epidermidis* was exposed simultaneously to both neuropeptides, and it was observed that CGRP and SP have antagonistic activities suggesting the existence of a common step in response of the bacterium to these neuropeptides. After exposure to CGRP, *S. epidermidis* is leading to an upregulation of interleukin 8 secretion by keratinocytes and RHE, suggesting that CGRP-treated bacteria are activating an inflammatory response. Then, the normal tolerance of the skin to *S. epidermidis* should be decreased. Because in keratinocytes an inflammatory response is usually associated with the secretion of antimicrobial peptides (39), the production of cathelicidin LL37 and human β defensin 2 (HBD2) by keratinocytes was investigated. However, a limited increase in LL37 secretion and a decrease in HBD2 were observed, suggesting that the effect of CGRP on *S. epidermidis* is not mediated by antimicrobial peptides (38). Analysis of the secretome of CGRP-treated bacteria did not reveal significant variations in the production of known virulence factors. In fact, CGRP appears to modulate the surface properties of *S. epidermidis* as CGRP-treated bacteria showed increased adherence to keratinocytes and reduced internalization and biofilm formation activity. These changes appear be associated and may be attributed to an increase in surface hydrophobicity (38).

### CGRP Sensing in *S. epidermidis*

**Figure 2:**
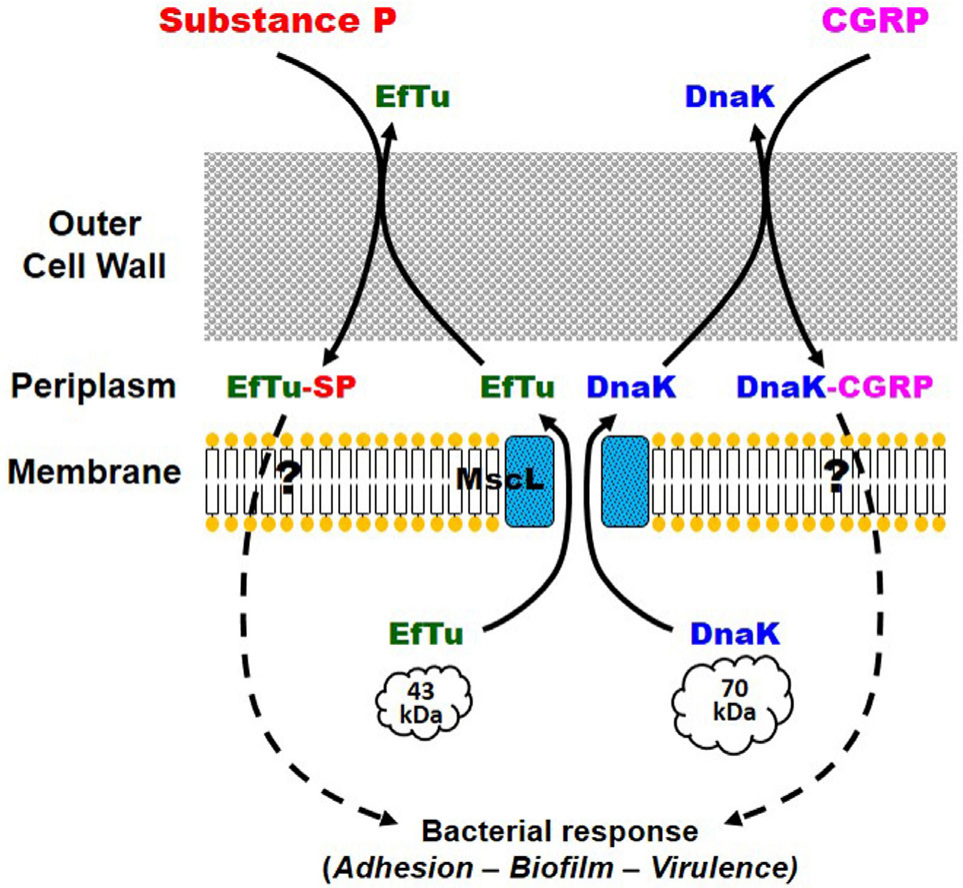
**Potential mechanism of action of substance P (SP) and calcitonin gene-related peptide (CGRP) on *Staphylococcus epidermidis***. EfTu and DnaK, bacterial SP and CGRP sensor proteins, respectively, are exported in the periplasmic area through the large mechanosensitive channel MscL. In the periplasm or in the bacterial microenvironment, EfTu and DnaK are, respectively, binding to SP and CGRP. Following a mechanism potentially close to that of bacterial quorum-sensing factors, but that remains to be identified, EfTu-SP and DnaK-CGRP should trigger the bacterial response including increased adhesion to the target cell, biofilm formation, and expression of virulence factors.

Investigating the CGRP sensor in *S. epidermidis* was leading to the identification of the chaperone DnaK as a specific CGRP binding protein in this bacterium (38) (Figure 1C). In *Staphylococci*, this protein related to the heat shock protein Hsp70 is known for its multiple functions in environment and stress adaption (40) and was found as a receptor for endothelial cells (41). Moreover, DnaK is showing sequence similarities with the CGRP receptor component protein (CGRP-RCP), a subunit of the eukaryote CGRP receptor that is required for signal transduction (42) (Figure 1D). Altogether these data suggest that DnaK is another member of the moonlighting proteins family. Moreover, a functional relation has been shown between DnaK and EfTu, the SP sensor. Indeed, in *E. coli*, DnaK and EfTu are translocated through the inner membrane by the same large mechanosensitive channel MscL (43). The MscL channels are also expressed in the membrane of *S. epidermidis*, and it was observed that gadolinium chloride, an inhibitor of mechanosensitive channels’ activity, was blocking the response of the bacterium to CGRP (38). Complementary studies based on a Δ*mscL* mutant confirmed the involvement of MscL in the response of *S. epidermidis* to CGRP (44). These observations were leading to the hypothesis that the export of DnaK and EfTu through MscL is required for the response of the bacterium to CGRP and SP (38). The initial induction of this process remains a major question as the neuropeptides appear acting both as ligands and signals for the export of DnaK and EfTu. However, it was noted that, even in the absence of stimulation, a limited amount of EfTu (and presumably DnaK) is present in the periplasmic area of *S. epidermidis* (38), and this pool of proteins should be sufficient for the initial activation step and induction of the export process. In addition, such ionic channels were first characterized as sensors of membrane mechanical stresses, regulating especially the cell turgor. It could also be conceivable that the interaction of some neuropeptides with the bacterial membrane would lead to cell wall mechanical perturbations, and consequently to the activation of MscL and the export of EfTu and DnaK (Figure 2).

## Involvement of Other Skin Neuropeptides

As mentioned in Section “Introduction,” many neuropeptides are produced and released in skin but only a few have been evaluated for their potential effects on cutaneous bacteria. Neuropeptide Y, somatostatin, and even POMC-related peptides have been shown to modulate the growth and virulence of bacteria, but until now, not in the context of skin physiology (12). A particular attention should be given to natriuretic peptides produced locally by endothelial cells that have been studied for their effects on Gram-negative bacteria such as *Pseudomonas*. Indeed, capillary vessels are present in abundance in skin, and especially in a region of the hair follicle, the bulge, at the vicinity of local bacterial populations. Natriuretic peptides, and especially the C type natriuretic peptide (CNP), have been shown to modulate the virulence and adhesion properties of *P. aeruginosa* (45), a common cutaneous opportunistic pathogen, and *P. fluorescens* (46), a member of the commensal microflora. The CNP bacterial sensor was recently identified in *P. aeruginosa* as another moonlighting protein, the amidase AmiC, an ortholog of natriuretic peptide receptor C (47). In fact, cutaneous bacteria should integrate the signals from a multitude of skin neuropeptides and in response adapt their physiology and virulence. Such local effects are also suspected in the case of a bacterium such as *Propionibacterium acnes* whose acneic strains are particularly virulent *in situ* but appear without significant virulence when they are grown *in vitro* (48).

## Conclusion

The physiological meaning of these observations and the reasons for the preservation or emergence of sensor systems for skin neuropeptides in cutaneous bacteria remain hypothetical. However, it appears nowadays that in gut, bacteria use hormones and neurotransmitters as signals for switching from a commensal to a pathogen behavior (49). It is interesting to note that, in skin, SP shows important variations of local concentration under the effect of pain, stress, or infection (15, 50, 51) and even in case of nervous breakdown (14). Then, local changes of SP concentration in skin, and presumably CGRP or other peptides, could trigger an increase in the virulence of cutaneous bacteria. These studies give consistence to the existence of a link between the central nervous system, the cutaneous microbiota, and skin homeostasis. Different products, including a polysaccharide rich in rhamnose and a thermal water of particular ionic composition, were found to be able to antagonize the effects of SP on *B. cereus, S. aureus*, and *S. epidermidis* (24). Dermocosmetic products have been developed nowadays on the basis of these observations, which also represent a new strategy for clinical applications in dermatology.

## Author Contributions

MF wrote the review and designed the figures. MF, OM, and P-JR realized blasts and proteins 3D views. VB, AG, AN, JE, P-JR, VP, SC, and OL revised the manuscript.

## Conflict of Interest Statement

The authors declare that the research was conducted in the absence of any commercial or financial relationships that could be construed as a potential conflict of interest.

The handling Editor declared a shared affiliation, though no other collaboration, with several of the authors AN, AG, VB, JE, P-JR, SC, OL, MF and states that the process nevertheless met the standards of a fair and objective review.
